# How Microgels Can Improve the Impact of Organ-on-Chip and Microfluidic Devices for 3D Culture: Compartmentalization, Single Cell Encapsulation and Control on Cell Fate

**DOI:** 10.3390/polym13193216

**Published:** 2021-09-23

**Authors:** Simona Argentiere, Pietro Aleardo Siciliano, Laura Blasi

**Affiliations:** Institute for Microelectronics and Microsystems IMM-CNR, Via Monteroni, University Campus, 73100 Lecce, Italy; simona.argentiere@gmail.com (S.A.); pietroaleardo.siciliano@cnr.it (P.A.S.)

**Keywords:** organ-on chip, 3D culture, cell-laden microgels, microencapsulation, compartmentalization, single-cell encapsulation, cell fate, stem cells

## Abstract

The Organ-on-chip (OOC) devices represent the new frontier in biomedical research to produce micro-organoids and tissues for drug testing and regenerative medicine. The development of such miniaturized models requires the 3D culture of multiple cell types in a highly controlled microenvironment, opening new challenges in reproducing the extracellular matrix (ECM) experienced by cells in vivo. In this regard, cell-laden microgels (CLMs) represent a promising tool for 3D cell culturing and on-chip generation of micro-organs. The engineering of hydrogel matrix with properly balanced biochemical and biophysical cues enables the formation of tunable 3D cellular microenvironments and long-term in vitro cultures. This focused review provides an overview of the most recent applications of CLMs in microfluidic devices for organoids formation, highlighting microgels’ roles in OOC development as well as insights into future research.

## 1. Introduction

Organ-on-chip (OOC) devices are in vitro miniaturized multicellular systems with defined architectures that are aimed at mimicking organs or tissues for biological/drug testing studies or tissue transplantation purposes [1,2]. In the past decade, OOC has emerged as in vitro miniaturized model that recapitulates the biochemical, mechanical, structural, and functional features of human organs by mimicking the in vivo-like cellular microenvironment. Although this model can potentially improve the prediction capability of preclinical studies in comparison to in vitro tests and animal models [3,4,5], the successful transition from conventional 2D cell culture to human OOC implies the development of microfluidically supported 3D architectures to mimic the native extracellular matrix (ECM), to induce cell-ECM and multicellular interactions, as well as to modulate many cell functions including polarity, morphology, and motility [6,7,8].

In this regard, there are many strategies to create 3D environments on a microdevice that have been recently reviewed, namely suspension, hydrogels, paper-based culture, and fiber scaffolds. Although they all have distinct advantages and applications in microfluidic devices, the hydrogels are particularly attractive for their potentiality in mimicking the ECM structure and composition [7]. Accordingly, there is a growing interest in the development of OOC platforms integrating hydrogels and microscale hydrogel particles (microgels) to build 3D microenvironments for cell culture with defined properties. While a large body of research has been devoted to hydrogels integrated into OOC platforms and recently reviewed, [8,9] this work is focused on the most recent applications of microgels in OOC and microfluidic devices for organoid formation. Since hydrogels are 3D networks of crosslinked hydrophilic macromolecules, they possess the ability to retain high amounts of water (ca. 90 to 99%) [10] and offer several advantages, such as free diffusion for small molecules, temperature sensitivity, relatively low cost, and ease of production [11]. Therefore, hydrogels represent valuable tools to build 3D microenvironments for cell culture in OOC devices and mimic the ECM due to their high biocompatibility and tunable properties, such as permeability, elasticity, stiffness, and chemical reactivity, while guaranteeing spatial organization of different cell types that allows cell–cell and cell–ECM interactions [3].

Several hydrogels have been proven to facilitate organoids formation [3,8,12,13], however, they pose challenges for convenient integration in microfluidic devices due to the lack of injectability for covalently crosslinked hydrogel [11]. In addition, methods to chemically pattern and spatially define hydrogel materials on-chip require the use of microfabrication techniques that can be advanced, costly, and time-consuming [9]. Alternatively, a strategy for achieving microenvironmental control over chemical functionality and/or mechanical properties without complex chemistries and convenient on-chip integration is to use microgels [14].

Microgels for 3D cell culture are usually made of natural polymers, since they guarantee higher biocompatibility and milder gelation conditions with respect to synthetic polymer-based microgels [4]. In addition, they ensure tunable mechanical properties, easy visualization, and control over the transport of oxygen, nutrients, and metabolites [4], as well as enhanced chemical reaction time and detection time scale due to their high surface-to-volume ratio [11]. Besides ensuring cell expansion and aggregation, like traditional 3D in vitro culture methods (i.e., bioreactors and bioprinting), microgels can also produce cell aggregates or tissues with controllable size and morphology at high throughput [6], since they act as modular building blocks to fabricate tunable 2D and 3D architectures [3,15].

Many fabrication methods produce monodisperse microgels, providing several advantages including (a) formation of uniform cells’ aggregates that can influence the differentiation into specific lineages; (b) reliable comparison of data across different experiments; (c) tight control and reproducibility of microenvironment even in scale-up conditions (i.e., bioreactors) [15]. Although cell-laden microgels (CLMs) may require extended optimization to adjust the synthetic microfluidic setup (flow rate, viscosity, density of different phases, interfacial tension, surface chemistry, and device geometry), [9] they offer unique opportunities to confine cells at the same scale of organ functional sub-units as well as to encapsulate and study individual cells [16]. Overall, microgels combine the favorable hydrogel properties, such as high-water content, softness, and tunability with ease of manipulation on-chip and cell-scale dimensions, thus offering new opportunities and innovative functionalities in OOC devices. The application of CLMs for tissue regeneration [15,17] and stem cell-based therapy [18], as well as their fabrication methods [19], have been recently reviewed, however, a complete overview of OOC platforms integrating CLMs has not been reported so far. Considering that “organ-on-chip” is a recent terminology, the research has been extended on applications of microgels in microfluidic 3D cultures that represent the precursor technology for OOC.

In this brief review, we first discussed the encapsulation of cells into microgels (i.e., microencapsulation), highlighting how it represents the premise for most of the microgels’ functions within the OOC and microfluidic devices. Then, the main applications of CLMs in the development of OOC and microfluidic platforms, namely compartmentalization, single-cell encapsulation, and control on proliferation, polarity and cell fate were argued. Since the focus of this work is on organoids and micro-organs formation on-chip, we will not review non-structured 3D aggregates or spheroids that are the object of the recent works by Nguyen et al. [20]. Furthermore, the capability of microgels to encapsulate and release bioactive molecules in a controlled manner has not been considered. The reader interested in production methods and on-chip manipulation of CLMs is referred to the excellent review by Huang et al. [6], whereas microencapsulation strategies in microfluidics-generated microgels for 3D cell culture have been thoroughly reviewed by many authors [18,21,22,23,24,25].

## 2. Microencapsulation and Cellular Confinement 

The integration of microgels in on-chip cell culture can be achieved through two main strategies: cells can be deposited and cultured on the microgel surface or encapsulated into the microgel matrix [15]. Despite the first approach (cell deposition on microgel surface) is characterized by high controllability and ease of cell manipulation, it has also typical limitations of 2D cell culture, being unable to accurately mimic the in vivo microenvironment provided by the ECM. Differently, microencapsulation is an attractive technique for 3D cell culture, since the microgel polymeric chains enable a more accurate mimicry of the complex 3D networks of macromolecules composing the ECM. In addition, microencapsulation is more attractive than other microscale 3D cell culture strategies, such as microwells, hanging drops, and cellular microarrays, since it provides more physiologically accurate ECM microenvironments to better modulate cell behavior including morphology, proliferation, and differentiation [25].

There are many strategies for cell encapsulation into microgels for 3D cell culture, including electrospray, lithography, emulsification, and droplet microfluidics [18,21], the last one being the most potentially impactful technology [19] because it is very versatile and allowing for tunable control on size, shape, monodispersity, and uniform or core-shell microgels’ morphology [21]. More interestingly, droplet microfluidics has a great advantage that can be integrated on-chip [26,27], and in this regard, a further step towards complete integration of microfluidic microgel synthesis is the development of all-aqueous systems that offer good biocompatibility while avoiding washing processes [28,29]. Importantly, monodisperse sub-100-μm microgels have been recently synthesized without chemicals that are harmful to cells, using a miniature gas-liquid coaxial flow capillary device, and this could open new potentialities in microencapsulation and miniaturization of 3D culture system [30]. Furthermore, recent achievements in the synthesis of microfluidics microgels with anisotropic shapes including fibers and disks represent a considerable improvement for culturing cells that are sensitive to spatial heterogeneity, such as cardiomyocytes and neurons [19,31]. Here the synthetic strategies for CLMs are not discussed further, since they have been reviewed previously [18,21,22,23,24], the focus instead is on the opportunities provided by the encapsulation of cells at the microscale as well as current applications of CLMs in OOC devices.

The encapsulation of cells into the microgel matrix results in a cellular confinement that provides the microfluidic setup with a powerful tool for on-chip organoid formation. Indeed, spatially confined cells can be precisely organized in space to achieve the desired 3D geometry, thus enabling the formation of either tunable or compartmentalized cell constructs. Microgels and their hydrophilic polymer networks can also confine cells while allowing the free diffusion of chemicals, nutrients, and metabolites and this is advantageous for cell therapy and modular tissue engineering, to guarantee immunoisolation, precise cell administration, and enhanced cell retention at the transplantation site [32,33,34,35,36]. When used in oil suspension, the confinement of cells in microgels facilitates the accumulation of cell secretions, enzyme molecules, and their catalytic products and that is attractive for the study of cellular functions, stimulus response, and cell-cell interactions [23,37,38]. The controlled encapsulation of single cells in the confined 3D matrix of microgels opens powerful opportunities to unravel the intricate cell-signal interactions that occur in bulk cell culture and in vivo systems, and to provide insights for biological processes and high-throughput drug testing [39]. Finally, since microgels have dimensions comparable to organ functional sub-units, they provide room for engineering physical and biochemical cues with high control. Therefore the microencapsulation opens the way to cell differentiation in a well-defined microenvironment and self-organization of cells into micro-organs [3].

## 3. Applications of Microgels in OOC

The application of CLMs in OOC represents an attractive tool to realistically mimic tissue models and organs, that are typically built from repeated, microscale functional units [40]. Indeed, spatially controlled assembly of CLMs results in architectures that reproduce modular tissue constructs while monitoring the microenvironment of individual cell types, [14,41] and enable the signaling experienced by cells in vivo [1] Different synthetic and natural polymers are exploited in the development of 3D architectures [7,14,15,16], and for example, alginate has been widely used to this purpose, as comprehensively discussed by Takayama et al. [25].

The assembly of CLMs for 3D culture in tissue engineering and regenerative medicine has been widely studied and reviewed [15,22,40,42], however, CLMs have been recently applied for 3D cultures in microfluidic devices for resembling organoids using one single or more cell lines. Microgel-based 3D cultures in microfluidic devices using one single cell line have intrinsic limitations in mimicking the complexity of tissue microenvironments, therefore they have been explored mainly for spheroids formation [20] which is not the focus of this review. The co-culture of two or more cell lines within the microgel matrix recreates the complexity and heterocellularity of native tissues; however, to reduce the risk of cross-contamination, compartmentalization is required, as discussed in the next paragraph.

### 3.1. Compartmentalization

Living tissues have an intrinsic heterogeneity due to the presence of highly organized, different cell types in ECM components. Mimicking this structural complexity is a key point to faithfully fabricate tissue/disease models and micro-organs and achieve affordable results in fundamental biological studies, drug screening and toxicity assessment as well as tissue transplantation and cell therapy [40]. To this aim, there is the need to spatially pattern multiple cell types in biocompatible ECM matrices, i.e., to compartmentalize the microfluidic 3D culture [15] that can be achieved through different strategies, including lithography and 3D printing [4]. A promising approach to achieve compartmentalized structures is the employment of multiphasic biomaterials that can be obtained by bottom-up and top-down strategies, as comprehensively reviewed by Werner et al. [40].

**Figure 1:**
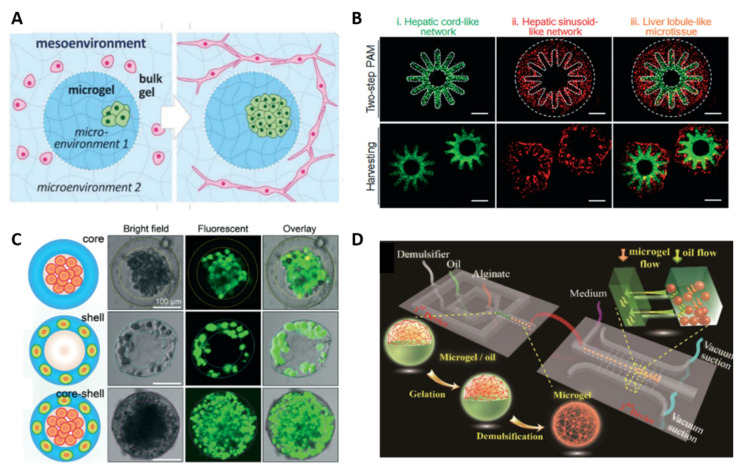
Various strategies to mimic the structural complexity of living tissues using compartmentalized CLMs. (**A**) Schematic of microgel-in-gel system which allows for adjusting micro- and mesoenvironmental parameters. Republished with permission of Royal Society of Chemistry, from Ref. [44]; permission conveyed through Copyright Clearance Center, Inc. (**B**) Radially orchestrated networks resembling (i) hepatic cords and (ii) hepatic sinusoids and their assembly in (iii) 3D liver lobule-like microtissue. Scale bars, 500 μm. Republished with permission of Royal Society of Chemistry, from Ref. [45]; permission conveyed through Copyright Clearance Center, Inc. (**C**) Assembly of different cells in the 3D core-shell microgel. HepG2 cells were confined in the core whereas NIH-3T3 fibroblasts were immobilized in the shell. The scale bars are 100 µm. Republished with permission of Royal Society of Chemistry, from Ref. [43]; permission conveyed through Copyright Clearance Center, Inc. (**D**) Schematic representation of microfluidic chip with two individual sub-devices: the first one is exploited for microgel droplet generation, in the second one it takes place the emulsification and the culture of cell-laden microgel under dynamic conditions. Reprinted with permission from Ref. [26]. Copyright 2019 American Chemical Society.

Microgels represent an attractive bottom-up strategy for compartmentalization, since they can be assembled in complex, multiphasic, and highly modular architectures without using complex chemistry. In addition, compartmentalization within the microgel structure can be achieved through chemical and stereolithographic approaches, providing room for the co-culture of different cell types while avoiding cross-contamination. Co-cultures can also be generated in microgels by using co-flow geometries where different cells are introduced at the droplet generation site. Core-shell “organ in a droplet” hepatic models are examples of structures that have been generated with this method [43]. The performance of the obtained microgel-based compartmentalized systems is a function of their components and combination and ensures improved functionality and complexity with respect to conventional single-phase systems [43]. Selected examples over the past five years are summarized in Table 1, whereas Figure 1 shows various strategies to mimic the structural complexity of living tissues using compartmentalized CLMs.

### 3.2. Single Cell Culture

Single-cell analysis has emerged as a powerful tool in biological and biomedical research for providing insights into the complex interplay between cell populations in vitro and in vivo [39]. Compared to larger hydrogels, microgels are more attractive model systems for studying cells at a single level, since they allow for efficient encapsulation of individual cells into a gel matrix with dimensions comparable to the cell size. As a result, cells experience an environment with a high surface-to-volume ratio that avoids mass transport limitations and consequently altered levels of oxygen, nutrients, and waste products [16,49,50,51], thereby enabling a long-term culture of encapsulated cells. Furthermore, microgels provide individual cells with a highly versatile, controllable, and reproducible microenvironment that allows them to be independently cultured, manipulated, and analyzed [39]. For this purpose, the number of cells per particle needs to be exactly controlled and this can be achieved by properly diluting the cell suspension or by adjusting the size of the droplets [21].

**Figure 2:**
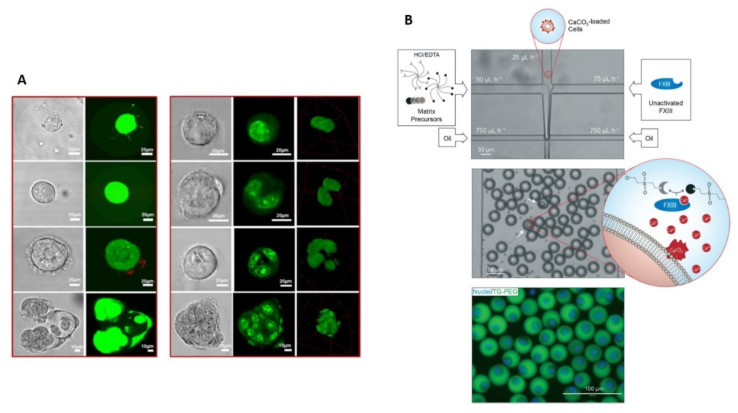
(**A**) Representative confocal microscopic images of cell growth of single MSCs within microgels. Live and dead cells were stained with Calcein and Ethidium Homodimer whereas alginate was labelled by the fluorescein. Reprinted from Ref. [53], Copyright 2020, with permission from Elsevier. (**B**) Single cell-laden 3D microgels mimicking stem cell niches in vitro (microniches) fabricated by droplet-based microfluidics. Matrix precursors, a diluted solution of cells loaded with CaCO_3_ nanoparticles, and unactivated Factor XIII (FXIII) are separately injected into a microfluidic chip. Reagents are joined in a laminar flow resulting in an emulsion for hydrogel formation. Cells, encapsulated in FITC labelled microniches, were stained with Hoechst 33342. Republished with permission of Royal Society of Chemistry, from Ref. [16]; permission conveyed through Copyright Clearance Center, Inc.

There are many reviews that focus on recent advancements in microgel-based 3D cell culture at the single-cell level. Zhu and Yang reviewed single-cell microgel based on natural and synthetic hydrogels, fabricated by droplet microfluidic techniques and cross-linked by various methods (i.e., chemical chelators, ultraviolet light, and temperature) for different applications, such as single-molecule/cell analysis or detection, single-cell sequencing, and molecular evolution [39]. Recently, Kamperman et al. reported single-CLMs fabricated by several techniques (i.e., droplet microfluidics, vibrating jet, inkjet, jet cutting, electrospraying, air-induced spraying) that offered unprecedented advantages, including high-throughput analysis of single cells, biomimetic 3D microenvironments, improved tissue engineering, and cell-based therapy [49]. Another review reports differences on single-cell microgel based on natural (agarose, collagen, hyaluronic acid, etc.) and synthetic (PEG and polyacrylate derivatives) hydrogels fabricated by extrusion-based single-cell and microfluidic-based droplet generation as promising tools to understand and direct complex biological systems and cell behaviors as well as enhanced in vitro cell culture, diagnostics, and screening and in vivo therapeutics [52]. The overview of the major developments on microgel-based 3D cell culture at single-cell level is listed in Table 2, whereas the most representative examples are shown in Figure 2.

### 3.3. Control on Proliferation, Polarity and Cell Fate

Although stem cells can differentiate into any type of cell in the adult body, it is often difficult to control microenvironmental factors that induce differentiation pathways. Stem cell fate is determined by intrinsic regulators and extrinsic signals. The physiological environment provides a perfect but complex combination of signaling, including the appropriate identity, abundance, location, and dynamics of stimuli that function in synergy with the intrinsic regulatory network to orchestrate the temporal-spatial control of self-renewal and differentiation. 

**Figure 3:**
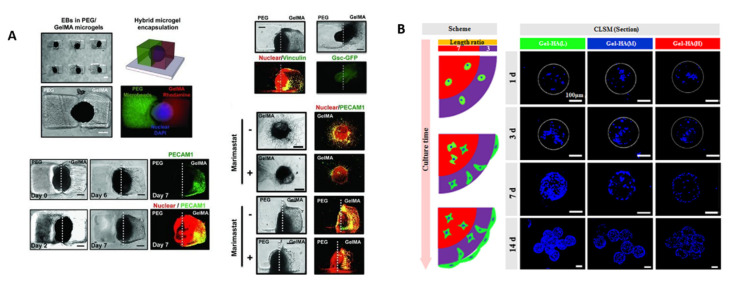
(**A**) Polarity induction in individual embryoid bodies (EBs) encapsulated in GelMA/PEG hybrid microgels with spatially patterned vasculogenic differentiation. All scale bars: 300 µm. Reproduced with permission from Ref. [57]. Copyright 2010 Wiley-VCH. (**B**) Bone marrow mesenchymal stem cell (BMSC) distribution in three gelatin/hyaluronic acid hybrid microgels with low, medium and high crosslinking densities. Reprinted from Ref. [58], Copyright 2020, with permission from Elsevier.

Microgels represent an attractive, scalable strategy for tailoring the stem cell microenvironment, since they can provide well-defined, uniform, and compartmentalized cell cultures. There are several reviews discussing the influence of the chemical composition of microgels on cell differentiation and polarization [21,55]; as an additional tool to control the cellular microenvironment, microgels can encapsulate and release in a controlled manner growth factors, as demonstrated by Siltanen et al. [56]. In Table 3 we summarize significative examples of CLMs for mimicking asymmetrical environments to investigate embryonic development or to direct polarization of cells, whereas Figure 3 illustrates two systems based on microgels that demonstrate how CLMs can provide polarization of embryoid bodies or mesenchymal stem cell differentiation.

## 4. Conclusions and Perspectives

The CLMs have become a valuable tool for 3D cell culturing and on-chip generation of micro-organs. The continuous advances in the synthetic strategies have led to the development of monodisperse CLMs with controlled size and chemical/physical properties as well as to precise compartmentalization. These microgels can resemble the functional organ sub-units and serve as building blocks to be assembled into 3D tunable tissue constructs. Overall, the microgels’ intrinsic features, such as high surface-to-volume ratio, spatial confinement, high tunability of both chemical/physical cues, and spatial assembly, make them ideal platforms to develop on-chip 3D tissue models that resemble cell–cell and cell–ECM interactions in a biomimetic microenvironment.

Despite these major improvements, several restrictions in the production methods of CLMs still remain and prevent their successful translation in clinics. First, current microencapsulation techniques lack tight control on the number of encapsulated cells per microgel and their centering within the microgel matrix. This leads to partial cell encapsulation and possible cell escape during microgel manipulation and 3D cell culture. Second, in the perspectives to move towards green chemistry approaches and to provide cells with more biocompatible environments, some attempts have been done on developing all-aqueous systems and removing fluorinated oils during microgels’ synthesis. However, this technology is still in its infancy and needs to be further studied and consolidated, for reliability assessment as well as for scale-up processes. Third, it must be considered that the ECM microenvironment has peculiar stiffness, permeability, and biochemical components composition, and all these parameters may dynamically change during time as a function of the growth stage. The CLMs still differ from the native ECM microenvironments and most microgels-based 3D cultures are static, while in vivo cells are exposed to mechanical stimuli that are typical of organ function. Since these physical and chemical stimuli can deeply influence the differentiation and self-organization of cells into functional tissues, efforts must be done towards physically and chemically dynamic 3D cultures, using molecular gradients and mechanical stimulation comparable to those experienced by cells in vivo.

## Figures and Tables

**Table 1:** Latest representative works of compartmentalized CLMs for micro-organs formation.

Ref.	Polymer	Synthesis	Application
[26]	Alginate	Chemical cross-linking within a multifunctional integrated microfluidic device	Study of cell−cell communications in a tumor-endothelial cell co-culture model
[29]	Alginate	(water/water) High-throughput droplet microfluidic system chemical cross-linking	Encapsulating rat pancreatic islets (β-TC6) for therapy
[38]	Alginate/gelatin	Droplet microfluidics using nonfluorinated oils and chemical cross-linking	Gastrointestinal niche exploiting crypt cells (as functional unit of the gastrointestinal tract) and Peyer’s patch cells (as functional unit of the immune system) to study intercellular interactions
[43]	Alginate	Chemical cross-linking of core-shell droplets within a flow focusing microfluidic device	“Organ in a droplet”: 3D liver model in a drop by controlled assembly of heterotypic cells in a 3D core–shell droplets
[44]	“Microgel-in-gel” based on poly(ethylene glycol)-heparin	Droplet microfluidics and crosslinking by Michael-type addition reaction	Modulation of micro- and mesoenvironmental parameters to reflect fundamental tissue properties or direct the maturation of 3D cell assemblies
[45]	Collagen, gelatin and agarose.	Pneumatic-aided micro-molding and physical gelation at 37 °C (collagen, gelatin) and 4 °C (agarose)	3D liver microtissue composed of a radially orchestrated network of hepatic cords and sinusoids
[46]	Methacrylic-gelatin based microgels covered by a secondary hydrogel overlay.	Multilayer printing technique and crosslinking by ultraviolet irradiation	Study of mutual influence on proliferation and migration in a co-culture system
[47]	Alginate	Chemical cross-linking within a flow-focusing microfluidic device	Pairing of single cells using multi-compartmental microgels for the study of cell-cell interactions
[48]	Collagen and gelatin	Electrostatic droplet method (collagen) and double emulsion (gelatin), using chemical and physical cross-linking, respectively	A biomimetic construction of bone tissue was realized using functional modules that mimic the osteon-like structures

**Table 2:** Major developments on microgel-based 3D cell culture at single cell level.

Ref.	Polymer	Synthesis Method	Application
[16]	TG-PEG hydrogel (polyethylene glycol precursors crosslinked by the transglutaminase FXIII)	Droplet microfluidics and enzymatic crosslinking	In vitro mimicking of stem cell niches (microniches)
[51]	Tyramine-conjugated polymers (dextran, hyaluronic acid)	Droplet microfluidics and enzymatic crosslinking	Preventing cell escape by cell centering to enable long-term culture and differentiation of stem cells
[53]	Alginate microgels	Droplet microfluidics and chemical crosslinking	Treatment of bone defects: osteogenesis and mineralization
[54]	Gelatin methacryloyl (GelMA)	Droplet microfluidics and gelation through ultraviolet irradiation	High-throughput analysis of single cells

**Table 3:** Significative examples of CLMs investigating embryonic development or polarization of cells.

Ref.	Polymer	Synthesis Method	Application
[59]	Natural or synthetic hydrogel	Bioprinting, photolithography, microcontact printing, microfluidics and chemical and photo-crosslinking	To model in vitro early stages of embryogenesis and gastrulation
[57]	GelMA/PEG	Combination of micromolding and photolithography techniques and photo-crosslinking	Polarization of individual embryoid bodies (EBs) with spatially patterned vasculogenic differentiation by encapsulating individual EBs inside microgels
[60]	PEG	Inverse suspension polymerization and chemical crosslinking	Macrophages can be encapsulated in microgel networks and polarized an inflammatory (M1) or anti-inflammatory (M2a) phenotypes
[58]	Gelatin/hyaluronic acid	Droplet microfluidics and crosslinking by Michael addition reaction	Mouse bone marrow mesenchymal stem cell (BMSC) proliferation, distribution and chondrogenesissyste

## Data Availability

Not applicable.
